# Where theory and practice of global health intersect: the developmental history of a Canadian global health initiative

**DOI:** 10.3402/gha.v7.23974

**Published:** 2014-07-24

**Authors:** Ibrahim Daibes, Sanjeev Sridharan

**Affiliations:** The Evaluation Centre for Complex Health Interventions, St. Michael's Hospital, Toronto, Ontario, Canada

**Keywords:** global health, equity, interdisciplinary research, complexity, research use, research excellence, theory of change

## Abstract

**Objective:**

This paper examines the scope of practice of global health, drawing on the practical experience of a global health initiative of the Government of Canada – the Teasdale-Corti Global Health Research Partnership Program. A number of challenges in the practical application of theoretical definitions and understandings of global health are addressed. These challenges are grouped under five areas that form essential characteristics of global health: equity and egalitarian North–South partnerships, interdisciplinary scope, focus on upstream determinants of health, global conceptualization, and global health as an area of both research and practice.

**Design:**

Information in this paper is based on the results of an external evaluation of the program, which involved analysis of project proposals and technical reports, surveys with grantees and interviews with grantees and program designers, as well as case studies of three projects and a review of relevant literature.

**Results:**

The philosophy and recent definitions of global health represent a significant and important departure from the international health paradigm. However, the practical applicability of this maturing area of research and practice still faces significant systemic and structural impediments that, if not acknowledged and addressed, will continue to undermine the development of global health as an effective means to addressing health inequities globally and to better understanding, and acting upon, upstream determinants of health toward health for all.

**Conclusions:**

While it strives to redress global inequities, global health continues to be a construct that is promoted, studied, and dictated mostly by Northern institutions and scholars. Until practical mechanisms are put in place for truly egalitarian partnerships between North and South for both the study and practice of global health, the emerging philosophy of global health cannot be effectively put into practice.

As an interdisciplinary field of research and action (1, 2), global health poses a challenge to the prevailing norms and metrics by which excellence in research and in population health practice is measured. The interdisciplinary nature of global health (3) contributes to the difficulty in defining it but, more importantly, to difficulties in designing, implementing, and evaluating global health initiatives and creating conducive environments in academic and health institutions as well as among donors for its support (1).

Like the issues it deals with, global health is a complex field that draws from multiple research and practice disciplines within complex environments as it seeks to understand the root causes of health disparities and take action toward both improving health globally and redressing health inequities among and between populations (4).

The history of global health is anchored in international health, public health, and tropical medicine (1). The term ‘global health’ began to be used on a wider scale in the 1990s with the rapidly growing forces of globalization and the shifting of focus, away from controlling epidemics spreading across national boundaries, toward addressing the health needs of peoples across the planet (5), as well as toward more focus on equity among populations and addressing the root causes of ill health. As this shift has begun to take hold, a multitude of global health initiatives have emerged, albeit without being anchored in a common understanding or definition of what the term global health means or implies.

A number of donors, research institutions, and training institutions have devised their own definitions of global health (2), generally guided by their own understandings of what global health might be and to guide their own investments in this evolving area of research and practice. Some such definitions have been driven by a desire to distinguish global health from the fields from which it is emerging. Others have sought to describe idealism in global health. Little, however, is known about the practical challenges of applying such definitions in large-scale programming.

Koplan and colleagues proposed one such definition, which is increasingly being referenced in the literature. It was later adopted by the Canadian Academy of Health Sciences as the one to guide Canada's future investment in global health (6). It is the definition we adopt for the purposes of this paper: ‘global health is an area for study, research, and practice that places a priority on improving health and achieving equity in health for all people worldwide. Global health emphasises transnational health issues, determinants, and solutions; involves many disciplines within and beyond the health sciences and promotes interdisciplinary collaboration; and is a synthesis of population-based prevention with individual-level clinical care’ (1).

This paper aims to shed light on some key implementation challenges in global health, drawing on the practical experience of a Canadian global health initiative – the Teasdale-Corti Global Health Research Partnership Program. It draws parallels between key elements and characteristics of global health described in the literature, the theory of change of the Teasdale-Corti program, and practical challenges collectively experienced by 26 projects that were funded by the program between 2006 and 2013.

## Methods

The opinions expressed in this paper are those of the two authors and do not necessarily represent those of the Global Health Research Initiative (GHRI). They are based on a review of relevant current literature exploring the definition and scope of practice of global health, the results of an end of program evaluation led by one of the authors (SS) in collaboration with the lead program manager (ID). They are also based on reflective exploration of the experiences of all teams involved in the program. Literature on the definition and scope of practice of global health was collected from search engines PubMed, Scopus, Google Scholar, and Google using the keywords, global health, evaluation, theory of change, equity, excellence, and international health.

**Fig. 1:**
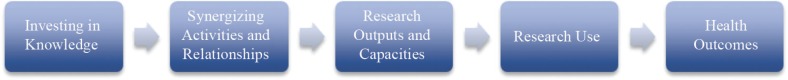
Simplified Initial Theory of Change of the Teasdale-Corti Program.

**Fig. 2:**
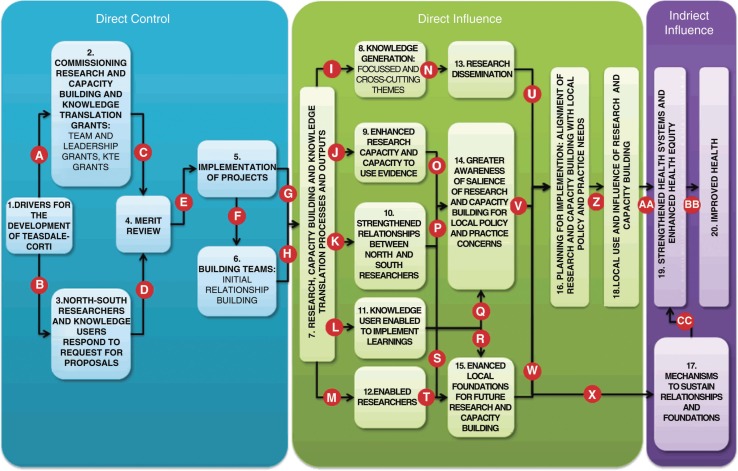
Detailed Theory of Change of the Teasdale-Corti Program.

Annual progress reports from all teams funded by the program were reviewed and analyzed by one author (ID) as part of ongoing progress monitoring with the aim of identifying implementation issues and challenges. Funding proposals and final technical reports were reviewed and analyzed by both authors in order to match the original intent of the teams with the final outcomes of their projects under the five broad components of the program's theory of change (Fig. 1 and Fig. 2).

## The Global Health Research Initiative

The GHRI (www.ghri.ca) is a research funding partnership between agencies of the Government of Canada responsible for health research and international development (The Canadian Institutes of Health Research, the International Development Research Centre (IDRC), Health Canada and the Department of Foreign Affairs, Trade and Development). It was created in 2001 in an effort to coordinate Canada's response to global health challenges. It aims to contribute to shaping the global health research agenda, influence policies relating to global health research, and to facilitate knowledge exchange among the partner agencies relating to global health practice. It did so through funding a suite of global health research programs involving Canadian and low- and middle-income country (LMIC) researchers and decision-makers (7).

## The Teasdale-Corti program

The $25 million flagship program of the GHRI aimed to take advantage of Canada's strengths to strengthen institutions in LMICs to undertake multi-year programs of research, capacity building, and action to address pressing LMIC health challenges of global significance (8).

The program consisted of three components: 1) Teasdale-Corti Team Grants, valued at approximately $1.5 million each, to enable teams of Canadian and LMIC researchers and research users to undertake programs of research, capacity building, and knowledge transfer and exchange, in response to particular LMIC health challenges; 2) Global Health Leadership Awards, valued at approximately $200,000 each, to enable mid-career researchers and decision makers from LMICs to advance their careers as global health leaders; and 3) a suite of strategic grants and awards aimed at facilitating learning about various aspects relating to global health, including capacity building, knowledge transfer and exchange (the use of research evidence to inform policy and practice), and ethics, in the context of global health. All grants were competitively awarded through a system of peer review.

The program emerged following commitment in a 2005 policy statement by the Government of Canada to an increase in its official development assistance with health as a key priority area and an emphasis on a knowledge-based approach to assistance to LMICs (9). It was designed to support efforts to bridge the health gap between rich countries and LMICs, with a realization that this gap is not only due to biomedical factors or the spread of pandemic diseases but also is exacerbated by global factors and determinants such as environmental degradation, inadequate and inappropriate social, health and education policies, ineffective national health and social services, and ineffective strategies for postgraduate and professional training; all of which are further exacerbated by inappropriate donor granting policies (7, 10).

The program was a bold attempt by the GHRI to challenge prevailing approaches to international development, international health, and the way public health research and practice are developed, implemented, and evaluated. It did so by emphasizing a three-pronged approach that incorporated research and action for understanding the upstream causes of ill health and inequity and for taking action on these causes, toward solving pressing LMIC health challenges with global significance. It also emphasized research capacity building as a mechanism not only to assist LMIC researchers and practitioners to take action to address LMIC health and equity challenges but also, as a Canadian initiative, to assist Canadian researchers to better navigate these contexts and operate as equal partners with their LMIC colleagues. The program was named in honor of Drs Lucile Teasdale and Piero Corti, a wife and husband team of physicians who dedicated their lives to healthcare in northern Uganda. The program ended in early 2013, but was followed by a second major investment by the GHRI, the $36 million ‘Innovating for Maternal and Child Health in Africa’ program, which draws on some of the lessons learned by the Teasdale-Corti program and adopts some of its features but with a much narrower thematic and geographic focus.

During the course of its 7-year implementation, the Teasdale-Corti program provided experiential evidence that attests to the validity of both GHRI's initial understanding of global health and the subsequent definition of the field offered by Koplan and colleagues (11). Of equal importance, the program and its large scale (spanning roughly 45 countries) also exposed many of the practical challenges to the implementation of global health initiatives that, if not noted and addressed, risk diluting this emerging area of research and practice and hampering its further development, maturation, and effectiveness in addressing highly complex health challenges globally. The program particularly exposed issues of complexity, heterogeneity of pathways, long timelines of impact, the need for a fundamental change in the way health research is assessed and rewarded, and the risk of slippage in global health development should these issues not be taken into consideration and addressed. Issues of accountability (in the traditional sense of return on investment for the donor) featured significantly in the developmental history of the Teasdale-Corti program and should, therefore, be considered in light of the complexity and risk inherent in global health initiatives and their long timelines of impact, that generally do not match with the short donor funding cycles and their associated accountability and return on investment requirements.

The Teasdale-Corti program exhibited typical characteristics of a complex system (12). It consisted of multiple components, each operating independently but were interconnected with the others through the larger program. Each of the components was a complex system in itself, and existed within, and interacted with, larger complex systems. The program and its components were all continuously adapting to their environments and evolved in a non-linear manner. The outcomes of the program overall were never prescribed or predetermined. Neither were the pathways to achieving these outcomes.

Realizing the inherent complexity of the program, and to help facilitate learning, its initial theory of change, that is, the means and processes by which the program can bring about improvements in health, was by design kept fairly broad. It only provided guidance for the program managers and grantees to work together to interpret the initial theory of change as the program progressed and evolved within the specific contexts under which each component project existed.

The theory of change still exhibited key features of global health as later defined by Koplan and colleagues. It identified a number of components and connections to help explain the pathways by which a global health initiative could have an influence on the intended outcomes. Exploring these characteristics and connections in the theoretical understandings of global health is important for exploring the practical applicability of the philosophy behind this evolving field.

Figure 1 simplifies the initial theory of change – as envisioned by the program designers and used by the program during its implementation – for the purpose of highlighting the core elements, characteristics and linkages. A more detailed visualization of the programs theory of change was developed by the authors toward the end of the program to anchor and guide its evaluation (Fig. 2). This more detailed and refined theory of change was developed through a process of consultation and exploration between the two authors and with key members of the GHRI's governing body who contributed to designing the Teasdale-Corti program. It was also built on a review of program documentation.^1^

### Investing in knowledge

This component was critical because it was the part of the theory of change where there was the most direct control. The decisions made at this stage about program design and project selection set a series of activities into motion, which were theorized by the GHRI to lead to the desired health outcomes.

It was critical at this stage for the program to specify the type of knowledge it sought and the type of research it was seeking to support – a focus on programmatic research that sought to understand upstream modifiable determinants of health and equity rather than focusing on specific diseases or single research projects with narrow priorities. This component of the program's theory of change also corresponds with Koplan and colleagues’ understanding of global health as a field of research that emphasizes determinants of health.

### Synergizing activities and relationships

The combination of research, capacity building, and knowledge translation was the catalytic component of the program's theory of change. It was theorized by the GHRI that this process would not only produce better knowledge through research and outcomes that are more likely to impact health but also that it would be more effective than the way traditional research is conducted – producing additional benefits through capacity building and orienting research for use in health systems. The program viewed research and practice in global health as inseparable.

Similarly, bringing together people with different types of expertise was critical to generating the type knowledge necessary to address complex health issues and challenges. The complexity of the health challenges being addressed and their underlying causes necessitated an interdisciplinary programmatic approach.

### Research outputs and capacities

While capacity building is almost never cited as an important component of global health, practical experience from the program suggested otherwise. By virtue of being a relatively new and still evolving approach to research and practice, capacity building seemed a core element of global health. For the Teasdale-Corti program, capacity building was a key mechanism to not only support and enable southern students and researchers to successfully participate in global health initiatives but also to enable Northern researchers and students to better navigate LMIC contexts, differences and complexities on an equal footing with their LMIC partners, and thus contribute to the equity orientation of global health.

### Research use

The involvement of the knowledge user in the project teams was seen as critical because of an implicit assumption that their involvement would increase the likelihood that the research produced would be relevant to and used by decision makers in order to lead to changes in the health system. Knowledge users were considered to be the connection to the health system that is needed for the knowledge produced through research to have an impact on health outcomes. Similar to Koplan and colleagues’ definition, this emphasizes global health as an area of practice as well as an area of research.

### Health outcomes

Health equity was the ultimate outcome intended by the program. It was not only an important focus for the program and the pathways by which improvements in health are made but also the driver behind it. It was its ultimate goal. The program, however, realized that this is a longer-term goal, with a timeline which goes beyond its funding timeframe, and that is affected by multiple other inputs and factors.

## Where theory intersects with practice

While the definition of global health and GHRI's understanding of it as articulated in the program's theory of change reflected an important theoretical shift from earlier paradigms of health research and international health practice, it was the practical application of this notion of global health that proved challenging. The program introduced new concepts of research excellence that emphasized not only technical merit and depth of knowledge, but also breadth of knowledge, interdisciplinary approaches, capacity building, action and practice. This, to a large extent, alienated the program from prevailing paradigms of research excellence in many research institutions that emphasized pushing the disciplinary boundaries of science rather than using the breadth of knowledge to solve practical health challenges.

An important challenge for the program – being primarily a funding mechanism – was that much of its implementation fell outside of the scope of its direct control. Once projects were selected through peer review, their implementation could only be influenced through ongoing monitoring and evaluation. Their pathways and timelines were vastly heterogeneous and lengthy, as well as very much affected by their specific contexts. The degree to which they achieved the program's vision of global health was also quite varied.

In order to explore how the program developed and to highlight some of the implementation challenges it faced, we divide it into two phases: the initial planning phase, and the implementation phase. The initial planning phase involved developing the initial program theory of change and translating that into competitive calls for proposals, peer review and selection processes, and setting up contractual granting arrangements with grantees. The implementation phase spanned the duration of all grants that were supported by the program. The key players during this second phase were the program's grantees.

## Initial planning phase

This was a critical phase in the development of the program and for its success. It shaped its contours as a global health initiative. The two critical elements in this phase were the calls for proposals and the peer review processes, which led to the selection of the projects that eventually made up the program. Both elements needed to clearly reflect the program's orientation as described in its theory of change. During this phase, the program attempted to operationalize its theoretical understanding of global health. Equity, egalitarian North–South partnerships and the global conceptualization of health were operationalized by bringing in experts from multiple LMICs to participate in and lead peer review deliberations thus allowing for Southern perspectives to prevail. Capacity building, research use and interdisciplinarity were operationalized by allowing students and junior researchers and practitioners to observe and contribute to peer review processes; including policy-makers and practitioners in peer review and selecting peer reviewers that spanned a wide range of research and practice disciplines.

This was a new type of process for many participants and was particularly challenging as it proposed different metrics for assessing research excellence that gave equal weight to research use and capacity building as it did to technical scientific merit; two criteria often ignored by traditional research funding. However, in order for the program to fund a portfolio of projects that met its understanding of global health – integrating study, research and action – this three-pronged approach was essential. This meant that some of the most technically meritorious proposals were not recommended for funding by peer review committees because they did not place sufficient focus on research use, action, practice and capacity building.

What distinguished this phase was that the competitions were conducted in two stages, an open call for expressions of interest, which led to closed, by invitation, requests for full proposals. During the second stage, there was considerable interaction between the program managers and grant applicants whereby program managers offered guidance and advice about the intended orientation of the program. This led to better alignment between the grant proposals and the program's objectives. As a result, while the success rate at the expression of interest phase for the Team Grant competition was approximately 15% (39 out of 259 expressions of interest), the success rate at the full proposal stage reached approximately 36% (14 grants out of 39 invited proposals).

Despite this approach, and while applicants offered convincing arguments about the involvement of knowledge users in the proposed projects, the portfolio of projects funded by the program continued to be more research than action oriented. This was a reflection of the fact that the majority of proposals were led by researchers rather than practitioners and the fact that the program itself was designed and administered by agencies that are anchored in the realm of research. Additionally, while knowledge users were engaged in the peer review processes, the deliberations of the peer review committees were more affected by the views of researchers than those of knowledge users by virtue of the researchers being more experienced with peer review than knowledge users and generally took the lead.

## Implementation phase

The implementation phase of the program started with the grant agreements being signed by the granting agency working on behalf of GHRI (the IDRC) and the various home institutions of the principal investigators. These memoranda of grant conditions were very broad in nature, only specifying high-level goals and objectives as well as timelines. They marked the end of the phase of direct control of the program and ushered in a new type of relationship between the program managers and the projects.

Unlike other Canadian granting agencies, IDRC employed an approach to research funding that entails direct engagement of program managers with grantees in the design, development and execution of projects toward a common research and development agenda. This approach was critical to the development of the Teasdale-Corti program. It allowed the program managers to engage with the project leaders to facilitate the progression of the projects toward achieving the program's common goals. This approach, however, was particularly alien to some grantees who viewed the program managers as intervening in their projects rather than facilitating their progression toward a larger common global health agenda which also incorporated learning from each project.

The following highlights some of the key challenges to the practice of global health as experienced by the program. These challenges are grouped according to the different components of the program's theory of change.

## Investing in knowledge

While all projects funded by the program subscribed to the global conceptualization of health (i.e. the goal of health for all people worldwide), with few exceptions the short funding timeline of the program made it difficult to consider the global implications of their work beyond the country, countries or regions where they operated. The two notable exceptions included one project which sought lessons from the successes and failures of comprehensive primary health care globally, and another which addressed pediatric pain management in northern Thailand hospitals and eventually contributed to the establishment of the Child Kind Initiative, modeled after UNICEF's Baby Friendly Hospital Initiative.

The primary challenge to operationalizing this concept stemmed from a seeming clash between the necessarily long timeline of impact of global health initiatives and the short timelines of accountability in public funding and its associated general focus on return on investment. While at its outset the Teasdale-Corti program was very clear about its global orientation, growing focus on return on investment and particularly its benefit to Canadians, being a Government of Canada funded program, caused some slippage in its global orientation and some regression back toward the international health orientation, the one from which the concept of global health had emerged in recent years.

Each project consisted of multiple interlinked sub-projects, which together aimed at contributing knowledge about different aspects of a health issue or challenge. The program's overall orientation and that of its component projects were firmly oriented toward addressing upstream modifiable determinants of health. Projects were able to do this by attempting to adopt interdisciplinary, systems approaches to research, that is, by combining and thinking across traditional academic boundaries in order to solve complex problems. This allowed them to consider health challenges and solutions within the wider systems and higher-level determinants. This approach, however, was most effectively applied by those projects that were ecohealth oriented, primarily because of their prior experience with interdisciplinary approaches – Ecohealth is a growing field of research, education and practice that addresses health and environmental issues arising from the interaction of societies and ecosystems (13). The majority of teams, however, ran into challenges in applying interdisciplinary approaches to research which, in order to effectively address complex health system challenges, entail stretching and working across academic disciplinary boundaries. Rather, they demonstrated a multidisciplinary approach whereby team members worked largely within their own disciplinary boundaries on specific components of a larger challenge which made them less effective in understanding and unpacking the complexity of the health challenges in question.

## Synergizing activities and relationships

The notion of global health as an area of research and practice was fundamental to GHRI's vision and to its understanding of research excellence. The program viewed research and practice as interconnected components of global health. It was not sufficient to conduct research solely for inquiry's sake and thus ignore the purpose of research to lead toward action to address complex health challenges. Research that does not incorporate action does not meet the basic standards of global health excellence.

This notion went directly against the ethos and metrics of research excellence prevailing in many academic institutions that participated in the program. By promoting action as an integral component of the research process, and by not placing as much emphasis on publishing for example, the program essentially undermined the systems under which Northern researchers – and many Southern researchers – operated in which creating new knowledge through research and publication is seen as the means for scientific and professional advancement. The program emphasized the application of knowledge rather than solely the creation new knowledge.

## Research outputs and capacities

As an evolving and maturing area of research and practice, research capacity building is another essential component of global health, to help create a core community of global health researchers and practitioners with a common vision and understanding of its scope of practice.

An important challenge that faced teams in this regard was related to establishing the right balance between research excellence and capacity building. The Teasdale-Corti program emphasized a high level of technical excellence and engaged highly competent researchers and practitioners. At the same time, the program also focused on building research capacity through training and mentoring. This necessitated stretching the timelines of most projects and, might have contributed to reducing the publication output of senior researchers who had to dedicate considerable time and effort to capacity building. The tension between the drive for technical excellence in research and the need for capacity building was not resolved by the program as many project teams felt they needed to make tradeoffs.

While mentoring proved to be a very effective approach to research capacity building, post-graduate training was less so. The short funding timeline of the program hampered both approaches. Mentoring for mid-career researchers and practitioners significantly stretched the timelines of all projects to the extent that all projects required time extensions of between 6 and 24 months beyond their original 4-year grants. This was still insufficient for many post-graduate students to complete their studies.

## Research use

In order to encourage research use and application, the program made it a requirement that knowledge users (decision makers in government, community groups, non-governmental organizations, etc.) be members of every team and participate in the design and implementation of every project. This approach was very much influenced by the knowledge transfer and exchange model first pioneered in Canada by the Canadian Health Services Research Foundation (CHSRF).

While it intended to forge a shift toward research use and practice, the program was still anchored in the realm of research and knowledge generation. While knowledge users were incorporated at the outset in every project, considerable slippage occurred as the various projects progressed. Being led by researchers rather than knowledge users, projects were generally not keenly aware of, or sensitive to, the realm of decision making and practice. As projects progressed, the involvement of knowledge users generally decreased in a number of projects. This was affected by a number of factors:

1. A general misconception about the pathways of knowledge use. Several projects confused knowledge transfer and exchange with research dissemination and therefore continued to operate within the realm of traditional health research rather than progressing toward global health research that incorporated practice.
2. Insufficient understanding of the realm of decision making, particularly in government, resulted sometimes in the recruitment of knowledge users who might not be in the best position of authority to effect change and integrate research with policy and practice.
3. While theoretically, the program emphasized action, the practical departure point of the program overall was that of research. Its competitive nature and peer review processes were mostly anchored in the research realm which was somewhat alien to those in the practice realm. The project leaders were mostly researchers and, consequently, many knowledge users assumed secondary roles.
4. At a higher level, while the program was that of the GHRI, its funding came only from the two research-focused members (the IDRC, which also administered the program, and the Canadian Institutes of Health Research). The secondary participation in the program by Health Canada and the Canadian International Development Agency (the two practice-oriented partners of GHRI) might have helped gear the program more toward the research side.
5. By focusing on the process realm, that is, specifying processes for projects to follow such as making the involvement of decision makers one of the mandatory criteria for project selection, and not focusing on the problem and solution realm, the program might have missed an opportunity to better experiment with the notion of global health being an area of research and practice. Similarly, the knowledge transfer and exchange model which the program adopted was challenged in some LMICs by their political and socioeconomic contexts, which are very different from the Canadian context where this model was first pioneered.

Similarly, on the higher program level, the long timeline of impact of the program and its component projects resulted in some slippage from the initial focus on finding long term and sustainable solutions to LMIC health challenges toward a focus on more immediate outputs and outcomes, such as publications and other immediate and short term benefits. This shift in focus was primarily dictated by some changes in focus by the program's parent organization.

## Health outcomes: equity as the principal driver of global health

Contributing to efforts to redress health inequities was a key driver of program. However, this was well beyond its scope of direct control or direct influence. The program could only indirectly affect health equity through the various projects it supported. The very long timelines of impact of the projects made it impossible for the program to directly assess its influence with this regard. Instead, the program operationalized the notion of equity by supporting and encouraging egalitarian partnerships between Canadian researchers and practitioners and their LMIC counterparts. This was built into the program's calls for proposals as well as the peer review and selection processes. Equitable partnership between North and South was fundamental to the program's vision of global health and represented a very significant shift from the international health paradigm.

While all project participants subscribed to the principle of equity, applying it in practice presented significant challenges. A majority of these challenges stemmed from the way academic institutions operate and from the traditional accountability relationships that exist particularly between Canadian universities and donors.

Pre-existing power differences and dynamics between Canadian and LMIC institutions played an important role in undermining the equity orientation of the program. By virtue of their size and their experience with Canadian research granting mechanisms, the majority of projects were, by their choice, administered by Canadian universities, the financial and administrative bureaucracies of which were alien to the notion of equitable North–South partnership that the donor was promoting. Additionally, the donor's finance and grant administration systems favored lower-risk Canadian institutions over generally higher-risk LMIC grantees and placed less restrictions on Canadian institutions than it did on LMIC ones. This placed Canadian institutions in a position of advantage and power over LMIC ones and as a result contributed to undermining the equity notion that the program promoted.

Another challenge stemmed from the way by which ethics review processes were carried out. All projects funded by the program were required to undergo ethics review in the institution or country where the work was to be done and not necessarily in Canada, since the vast majority of the research was conducted in LMICs. However, all Canadian participants were required by their institutions to obtain ethics approval from their own institutions’ ethics review boards regardless of whether or not those same projects had already been reviewed and accepted by LMIC ethics review boards. This served to undermine fully accredited LMIC ethics review boards as well as the equity orientation that the program promoted.

While on the outside, project administration might not seem as a determinant of health equity, the fact that powerful Northern institutions were able to dictate their positions on the weaker LMIC institutions featured significantly throughout the Teasdale-Corti program and in many instances prevented the development of truly egalitarian partnerships as initially envisioned by the program.

## Discussion and implications for future global health initiatives

Both the definition of global health proposed by Koplan and colleagues and GHRI's understanding of it reflect an important theoretical and philosophical shift from the previous international health paradigm. From a practical perspective, however, the on the ground large scale practice of global health within this new paradigm poses some significant challenges. Global health attempts to change well-established and long-standing paradigms of research and practice as well as established metrics of excellence. The inclusion of action on the determinants of health as an integral component by which research excellence is assessed, and the focus on interdisciplinary approaches, make it difficult for global health research and practice to be easily acknowledged or rewarded in some academic institutions. In many instances, the interdisciplinary nature of global health makes it difficult to publish in certain journals, as work is deemed too broad to readily fit within certain disciplinary orientations or constraints. Global health's focus on breadth rather than depth of knowledge might further hamper its acceptance in mainstream academia.

In order for global health practice to achieve better success in its equity orientation, systemic and structural institutional hindrances to equity need to be acknowledged and addressed. Northern institutions in particular need to acknowledge the uneven power balance they have with Southern institutions. They need to develop a higher level of comfort with equitable partnerships. Donors play a critical role in helping to even the balance of power between North and South. Both donors and Northern academic institutions engaged in global health need to build equity with the South into their administration and finance systems and reorient these systems to work in partnership with their global health researchers and practitioners rather than against them.

While accountability, particularly financial accountability, might be paramount to public donors and to research institutions receiving public funds, not having some level of trust in Southern institutions and not accepting a higher level of risk in the accountability and return on investment sense has proven to be particularly detrimental to the practice of global health and its equity orientation. The notion of accountability in some Southern contexts has proven to be vastly different from the Canadian understanding of it.

The important role of the donor in shifting the paradigm from international health to global health was quite significant. The large size of the grants that the various Teasdale-Corti projects received caused many academic institutions to pay closer attention and offer more acknowledgment to those researchers, particularly in Canada who, through the program, brought fairly significant research grants to their institutions. In order, however, for this shift and increasing acceptance to continue to take hold, it is important that this fairly large program in global health not be a one off funding initiative and that the momentum it created to continue. While large in scale in Canadian health funding standards, the program still represented only a small portion of the total health research funding envelope in Canada and it would be important that funding for global health continue at least at the level that was initiated by the Teasdale-Corti program, and in the same philosophical direction.

The long timelines of impact of global health and its inherent complexity pose a particular risk to its development and maturation as a field. The short timelines of accountability and return on investment generally preferred by donors as well as the preference of some for simple and quick solutions to health challenges all create a push back from the emerging global health paradigm to that of international health where short-cycle aid by rich nations to poor nations rather than partnership, and simple immediate remedies to complex challenges rather than addressing complexity with complexity are the prevailing approaches.

While action on root causes of health challenges was a key component of GHRI's understanding of global health, this concept was very difficult to put into practice. The primary reason was that the program itself as a funding initiative supported by two research agencies and anchored in the research domain found it difficult to move from the research realm to the three pronged approach of research, practice and capacity building. There were few structural and procedural incentives to support the research, practice and capacity building paradigm that the program attempted to promote.

A lack of understanding of what a knowledge user is also contributed to the significant heterogeneity in achieving success in the research use realm. The initial procedural focus of the program also contributed to this as the program, prescribing a model of practice rather than seeking ideas that were anchored in the solution sphere, also contributed to the confusion by some participants between knowledge transfer and exchange and simple research dissemination through publications, meetings, and conferences. Had the initial calls for proposals been focused on seeking ideas for solving complex health challenges rather than demanding a particular model of engagement of knowledge users would have put the program in the solution sphere and might have generated more favorable results.

In order for global health to continue to grow, develop, and mature as an effective means to addressing health inequities and complex health challenges of global significance, important changes need to take place in health research granting systems, particularly allowing for risk taking and long timelines, and accepting complexity in health, health systems, health challenges, and solutions.

## Footnotes

1 An interactive representation of the detailed theory of change of the program can be found at http://torontoevaluation.ca/teasdale-corti/theory-of-change.html
